# Effects of eccentric contraction-based resistance exercise on physical performance in chronic stroke patients: A randomized controlled trial

**DOI:** 10.1097/MD.0000000000043491

**Published:** 2025-08-08

**Authors:** Younji Kim, Seunglyul Oh, Dae Young Kim, Jae-Young Lim

**Affiliations:** a Department of Rehabilitation Medicine, School of Medicine, Ewha Woman’s University Seoul Hospital, Seoul, Republic of Korea; b Research Institute, Hospi Co., Ltd, Hanam, Republic of Korea; c Senior Exercise Rehabilitation Laboratory, Department of Gerokinesiology, Kyungil University, Gyeongsan-Si, Gyeongsanbuk-Do, Republic of Korea; d Department of Rehabilitation Medicine, Seoul National University College of Medicine, Seoul National University Bundang Hospital, Seongnam, Republic of Korea; e Institute on Aging, Seoul National University, Seoul, Republic of Korea.

**Keywords:** function recovery, physical functional performance, resistance training, Stroke rehabilitation

## Abstract

**Background:**

Chronic stroke patients often experience muscle weakness, reduced mobility, and impaired physical function. Eccentric contraction-based resistance exercise has been proposed as an effective intervention to improve neuromuscular function and mobility; however, its effects remain unclear. This study compared the effects of eccentric overload resistance training using a flywheel device and traditional resistance training on physical performance and muscle strength in chronic stroke patients.

**Methods:**

This 8-week, randomized controlled trial included chronic stroke patients who were assigned to either an intervention group (INT; eccentric flywheel-based resistance exercise) or a control group (CON; conventional resistance training). Physical function was assessed based on the timed up-and-go test, 6-minute walk test, 5-times sit-to-stand test, gait speed, and isokinetic muscle strength before and after the intervention.

**Results:**

Of the 40 participants enrolled, 36 completed the study (INT group: n = 18; CON group: n = 18). Both groups showed improvements in physical function outcomes, such as gait speed, balance, and functional mobility, postintervention. Muscle strength also improved in both groups, with no significant between-group differences in functional or muscle strength outcomes. Subgroup analysis revealed additional benefits of eccentric training for participants with limited walking ability.

**Conclusion:**

Both resistance training approaches improved physical performance in chronic stroke patients. However, eccentric overload resistance training offered additional advantages in functional mobility, particularly for patients with severe impairments. Both eccentric overload and traditional resistance training significantly improved physical performance in chronic stroke patients, highlighting the importance of structured exercise in rehabilitation.

## 1. Introduction

Stroke is a leading cause of long-term disability, often resulting in permanent motor and sensory impairments that limit functional movements and regular physical activities.^[1]^ Among long-term stroke survivors, the most commonly reported challenges include mobility issues (58%), fatigue (52%), muscle weakness (45%), and falls (44%),^[2]^ primarily due to muscle atrophy, reduced motor unit recruitment, and impaired coordination between agonist and antagonist muscles.^[3,4]^ As a result, stroke survivors experience progressive physical decline, characterized by muscle degeneration, significant loss of muscle mass and strength, and increasing frailty. Exercise capacity is notably reduced, with survivors exhibiting up to a 50% lower capacity compared to age-matched healthy individuals, particularly during the subacute and chronic phases of recovery.^[5,6]^

Effectively addressing the neuromuscular changes seen in stroke patients requires a carefully tailored exercise intervention strategy. Traditional rehabilitation exercises have long been advocated for improving mobility, muscle strength, and functional recovery in stroke survivors.^[7–9]^ These conventional approaches typically focus on progressive resistance training to enhance postural control, mobility, and overall strength, demonstrating efficacy in facilitating physical recovery.^[9,10]^ However, such methods may fall short of addressing the needs of individuals with more complex complications resulting from central nervous system damage.^[9,11,12]^

Eccentric contraction exercises, which involve controlled muscle elongation under tension, promote muscle regeneration and neural adaptations while imposing a low cardiopulmonary load,^[13,14]^ making them suitable for high-risk populations such as patients with stroke.^[15–17]^ Recent studies highlight their benefits in improving muscle function and mobility in neurological conditions.^[8,18,19]^ Flywheel resistance training, known for its portability and ability to generate greater force than traditional weights,^[20,21]^ has shown efficacy in enhancing neuromuscular function and physical performance in stroke patients without worsening spasticity.^[5,22]^ Consequently, eccentric overload resistance training has emerged as a promising and effective rehabilitation strategy for stroke recovery. However, the evidence regarding the efficacy of eccentric training remains mixed. While some studies highlight its benefits for muscle mass and mobility,^[5,22,23]^ others report no significant advantage over traditional resistance training.^[24–26]^ This inconsistency suggests that although eccentric contraction-based interventions show potential, their superiority over conventional resistance training is not universally supported. Therefore, further research is warranted to elucidate their clinical impact, particularly in specific populations such as chronic stroke patients.

The primary aim of this study was to compare the effects of eccentric overload resistance training using a flywheel device with those of traditional resistance training in chronic stroke patients. This investigation assessed the impact of both interventions on physical performance and muscle strength, aiming to determine whether eccentric training provides distinct advantages over conventional methods for enhancing functional recovery and strength.

## 2. Methods

### 2.1. Study design

**Figure 1.**
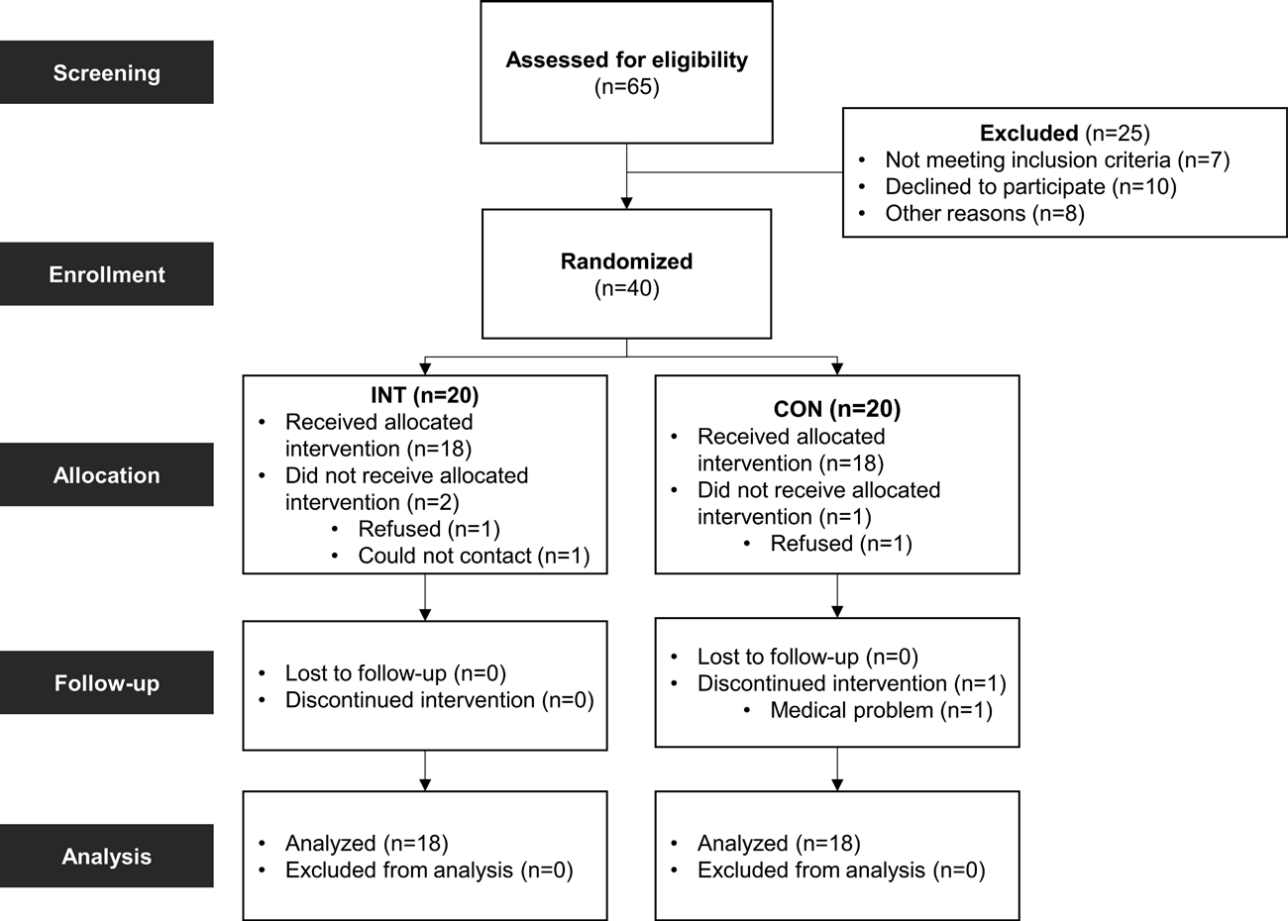
Consolidated Standards of Reporting Trials (CONSORT) flow diagram. Progress through the phases of the parallel randomized trial of both groups.

This study was an 8-week, single-blinded, single-center, parallel-group, prospective randomized controlled trial designed to evaluate the effects of eccentric overload resistance exercise on functional recovery in patients with chronic stroke. After obtaining written informed consent, potential participants were screened for eligibility. Eligible individuals were randomly assigned to either the intervention group (INT) or the control group (CON) in a 1:1 ratio. Participants completed an 8-week program and underwent assessments at baseline and after the intervention period. The study was registered at ClinicalTrials.gov (NCT04600050) and received ethical approval from the Institutional Review Board of Seoul National University Bundang Hospital (B-2006/616-006). All procedures adhered to the Consolidated Standards of Reporting Trials guidelines (Fig. 1).

### 2.2. Participants

Participants with chronic stroke were recruited from the Department of Rehabilitation Medicine at Seoul National University Bundang Hospital in South Korea between September 15, 2020, and March 30, 2021.

The study included individuals aged 50 years or older with hemiplegia, resulting from either ischemic or hemorrhagic stroke that occurred >1 year prior to the study, who were capable of effective communication, as evidenced by a score of 24 or higher on the Korean version of the mini-mental state examination (MMSE-K),^[27]^ and had a functional ambulation category^[28]^ score of 3 or higher, indicating partial to full ambulation capabilities. We excluded patients with brain lesions not caused by stroke, quadriplegia, conditions where exercise interventions were deemed inappropriate (e.g., uncontrolled hypertension, angina pectoris, or congestive heart failure), or fractures within the prior 6 months.

### 2.3. Randomization and blinding

Participants were randomly assigned in a 1:1 ratio to either the INT or CON group using a pre-generated randomization table, with allocations made sequentially as participants enrolled. While it was not feasible to blind participants due to the nature of the exercise intervention, a single-blind design was employed, ensuring that only the outcome evaluator was blinded to group assignments.

### 2.4. Sample size

The sample size calculation was based on a previous randomized controlled trial that examined the effects of eccentric-based training on physical performance, which is the primary outcome of this study.^[29]^ With an alpha level of 0.05 and a statistical power of 0.80, and considering the repeated measurements before and after the intervention, it was determined that 16 participants per group were needed. To account for an anticipated 20% dropout rate, a total of 40 participants were recruited.

### 2.5. Rehabilitation program

**Figure 2.**
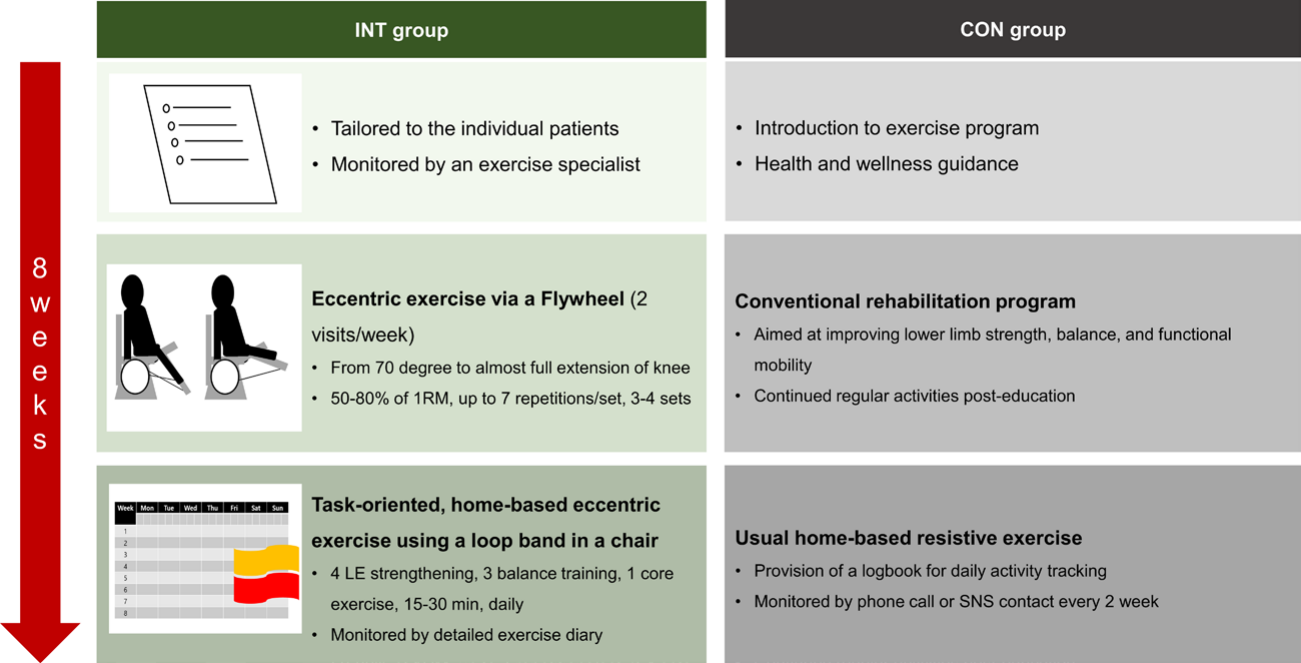
Rehabilitation protocol used in this study.

Participants completed either an eccentric flywheel-based resistance exercise program or conventional resistance training over 8 weeks, according to their assigned group. The rehabilitation exercise sessions were conducted by an exercise specialist under the supervision of a rehabilitation physician in the rehabilitation gym of a university hospital (Fig. 2). During the entire study period, no serious adverse events or significant side effects were observed.

### 2.6. INT group

Participants in the INT group followed a structured eccentric overload resistance exercise regimen. The rehabilitation exercise sessions were conducted in the rehabilitation therapy room. The primary exercise utilized a flywheel leg press (YoYoTM Technology AB, Stockholm, Sweden) and was performed twice weekly, with a minimum 48-hour rest period between sessions (5). Eccentric contraction loads were managed using the Flywheel app (SmartCoachTM, Stockholm, Sweden) and set at 50% to 80% of the participant’s one-repetition maximum, with a target rate of perceived exertion of 11 to 15. Each session began with a 10-min warm-up, which included light walking and dynamic stretching exercises focused on the lower extremities. The primary exercise consisted of leg presses performed for 3 to 5 repetitions per set, with a total of 3 to 4 sets. A 3-minute rest period was enforced between sets. Participants were instructed to maximize the flywheel’s rotational speed during the concentric phase and reduce it during the eccentric phase. Each repetition involved a maximal-effort extension–flexion movement, ranging from approximately 70° of knee flexion to near full extension. The eccentric load was optimized by resisting during the first third of the eccentric phase and applying minimal resistance during the remaining two-thirds, ensuring an effective load distribution while minimizing the risk of injury.

In addition to the flywheel exercises, participants followed a task-oriented, home-based program using a loop band 2 to 3 days per week. This supplementary program focused on strengthening, balance, and core exercises, all performed in a seated position. Eccentric contraction exercises emphasized slow movements during the eccentric phase and faster movements during the concentric phase. Exercise intensity and repetitions were adjusted based on the rate of perceived exertion scale, ensuring that the exercises were customized to each participant’s individual capacity and range of motion.

### 2.7. CON group

Participants in the CON group engaged in usual care and exercise education as part of a conventional task-oriented, home-based exercise program, aligning with standard rehabilitation practices for stroke survivors. This intervention lasted for 8 weeks. Following the education, participants continued with their regular activities and exercises as they would typically do in their daily lives.

During the initial evaluation visit, participants were provided with exercise and health education, which enabled them to perform traditional resistance exercises at home. These exercises were designed to enhance lower limb strength, balance, and overall functional mobility. Participants were instructed to perform 15 repetitions per exercise, progressing from 1 to 4 sets over time depending on their individual strength levels and response to the training. To ensure proper execution and adherence to the program, participants were provided with instructional videos, detailed written guidelines, and a logbook to record and manage their daily activities. Every 2 weeks during the intervention period, follow-up assessments were conducted through phone calls or social networking services to monitor physical activities and exercise adherence. Additionally, participants were encouraged to maintain their usual medical treatments and participation in general exercise programs offered at local medical centers and community health centers, and this was monitored throughout the study.

### 2.8. Outcomes

Baseline assessments included medical history, physical examination, body composition analysis, and functional ambulation category^[28]^ for mobility. Participants also completed questionnaires on function and disability, including the MMSE-K,^[27]^ modified Barthel index,^[30]^ and European quality of life 5 dimensions questionnaire (EQ-5D).^[31]^

Primary outcomes were assessed using various physical performance tests related to daily activities. Participants received detailed instructions and had the opportunity to familiarize themselves with each test procedure. The tests included:

#### 2.8.1. Timed up-and-go test

Measures functional mobility and balance by timing how quickly participants stand, walk 3 m, turn, and return to sitting. Shorter times indicate better mobility.^[32]^

#### 2.8.2. 6-minute walk test

Evaluates aerobic capacity and endurance based on the distance walked in 6 minutes, with longer distances reflecting better mobility.^[33]^

#### 2.8.3. Gait speed

Gait speed was assessed at a normal pace over an 8-meter walkway, with timing recorded over the middle 4 meters after allowing 2 m for acceleration and 2 m for deceleration. Faster speeds indicated better functional mobility and reduced fall risk.^[34]^

#### 2.8.4. Five times sit-to-stand test

Measures lower limb strength by timing 5 consecutive sit-to-stand movements, with shorter times indicating better strength and mobility.^[35]^

Secondary outcomes included muscle function assessments using isokinetic strength testing with a PrimusRS dynamometer (BTE Inc., Hanover) to measure isokinetic and eccentric muscle strength. This method provides precise evaluation of muscle force output, particularly relevant for eccentric strength assessment in stroke patients.

All variables were measured at 2 time points: before and after the 8-week intervention.

### 2.9. Data analysis

In this study, 40 participants were initially recruited; however, 4 withdrew – 2 declined participation, 1 could not be contacted, and 1 experienced a medical issue – leaving 36 participants for analysis. A complete-case analysis was used, assuming missing data were completely at random, with sensitivity analyses conducted to assess its impact.^[36]^

Baseline characteristics were compared using independent *t*-tests for continuous variables and chi-square or Fisher exact tests for categorical variables. Physical performance and muscle strength at baseline were also compared between groups using independent *t*-tests.

To analyze group × time interactions, repeated-measures ANOVA was performed, with Bonferroni correction applied for multiple comparisons (adjusted α = 0.0125). For the exploratory subgroup analysis of participants with limited walking ability (<400 m in the 6MWT), Wilcoxon signed-rank tests assessed within-group differences, while Mann–Whitney *U* tests compared between-group changes.

All analyses were conducted using SPSS (Version 27.0, IBM Corp., Chicago), with statistical significance set at *P* < .05, and Bonferroni correction applied where necessary.

## 3. Results

The 8-week intervention study included 36 participants, evenly allocated to the INT group (n = 18) and the CON group (n = 18). The flow of participants through the trial is illustrated in Figure 1.

### 3.1. Baseline characteristics

Table 1 summarizes the baseline characteristics of the participants. The mean age was 68.11 ± 7.15 years in the INT group and 64.00 ± 8.16 years in the CON group. No significant differences were observed between the groups in the gender distribution, body mass index, or waist-to-hip ratio. Similarly, baseline assessments of ambulatory function, functional independence, cognitive function, and quality of life revealed no significant differences. However, significant differences were noted between the groups in certain body composition and circumference measurements.

**Table 1:** Baseline characteristics of the participants.

Characteristics	INT (n = 18)	CON (n = 18)	*P*
Age (yr)	68.11 ± 7.15	64.00 ± 8.16	.117
Gender (male: female)	13:5 (27.8)	14:4 (22.2)	1.000
Height (cm)	163.72 ± 7.55	165.69 ± 7.20	.429
Weight (kg)	64.41 ± 9.40	70.88 ± 9.24	.045*
BMI (kg/m^2^)	24.00 ± 2.91	25.86 ± 3.22	.078
Duration of stroke (yr)	3.11 ± 3.64	3.33 ± 1.65	.815
Stroke type (n)			
Hemorrhagic stroke	4	6	.710
Ischemic stroke	14	12	
Waist circumference (cm)	87.11 ± 7.90	91.64 ± 6.97	.077
Hip circumference (cm)	93.78 ± 5.24	97.33 ± 4.79	.041*
Waist-hip ratio	.92 ± .05	.94 ± .05	.447
Thigh circumference (cm)			
Involved side	48.19 ± 3.46	51.54 ± 4.14	.013*
Uninvolved side	49.44 ± 3.91	51.88 ± 4.66	.099
Calf circumference (cm)			
Involved side	33.89 ± 2.26	36.81 ± 2.37	.001*
Uninvolved side	34.47 ± 2.22	36.89 ± 2.58	.003*
Body composition			
TFM (kg)	21.71 ± 6.50	23.57 ± 6.05	.380
PBF (%)	33.48 ± 7.64	33.09 ± 6.82	.871
SMM (kg)	22.85 ± 4.19	25.74 ± 4.05	.043*
ASM (kg)	18.07 ± 3.56	2.31 ± 3.27	.058
ASMI (kg/cm^2^)	6.67 ± .85	7.34 ± .72	.015*
FAC			
3	3	0	.105
4	1	0	
5	14	18	
MMSE	28.06 ± 1.59	28.61 ± 1.04	.223
MBI	97.72 ± 4.42	99.67 ± 1.03	.085

Values are expressed as mean ± standard deviation or number (%).

ASM = appendicular skeletal muscle mass, BMI = body mass index, CON = control group, FAC = functional ambulatory category, INT = intervention group, MBI = modified Barthel index, MMSE = mini-mental state exam, PBF = percent body fat, SMM = skeletal muscle mass, TFM = total fat mass.

* *P* < .05.

### 3.2. Baseline physical function and muscle strength

As presented in Table 2, baseline evaluations indicated that lower extremity strength was significantly lower in the INT group compared to the CON group. However, no significant differences were observed in physical performance assessments between the groups, except for the TUG test.

**Table 2:** Initial evaluation of physical performance and muscle strength of the participants.

		INT (n = 18)	CON (n = 18)	*P*
Physical performance				
TUG (s)		16.89 ± 14.48	8.77 ± 2.12	.030*
6mWT (m)		374.67 ± 167.70	462.06 ± 78.03	.056
Gait speed (m/s)		1.04 ± .47	1.28 ± .22	.057
5TSTS (s)		18.01 ± 13.30	11.59 ± 3.66	.063
>400 m walking ability		10 (55.6)	13 (72.2)	.489
Lower extremity strength				
Isok 60 d/s E ST (pt)	I	54.89 ± 17.57	81.89 ± 22.11	<.001*
	UI	72.17 ± 15.69	89.33 ± 2.63	.008*
Isok 60 d/s F ST (pt)	I	43.00 ± 16.05	58.50 ± 16.50	.007*
	UI	48.78 ± 17.32	62.22 ± 14.51	.017*
Isokinetic End (%)	I	38.86 ± 17.59	53.07 ± 22.63	.043*
	UI	45.16 ± 19.06	62.78 ± 21.44	.014*
Ecc ST (pt)	I	85.8. ±24.64	113.61 ± 24.66	.002*
	UI	92.78 ± 26.11	119.44 ± 19.39	.002*

Values are expressed as mean ± standard deviation or n (%).

5TSTS = 5-times sit-to-stand test, 6MWT = 6-minute walk test, CON = control group, Ecc ST = eccentric contraction strength, I = impaired side, INT = intervention group, Isok 60 d/s E ST = Isokinetic 60 degree/sec extensor strength, Isok 60 d/s F ST = isokinetic 60 degree/sec flexor strength, Isokinetc End = isokinetic muscle endurance, TUG = timed up-and-go test, UI = unimpaired side.

* *P* < .05.

### 3.3. Physical performance assessment

As shown in Table 3, significant improvements were observed in both the INT and CON groups over the 8-week intervention period across various measures of physical performance. There was a main effect of time, with statistically significant enhancements in all functional outcomes, including the TUG, 6MWT, gait speed, and 5TSTS test, between the pre and postintervention assessments.

**Table 3:** Primary outcome variables before and after the interventions.

		INT (n = 18)	CON (n = 18)	Group*time interaction *F* (*P*)	Main effect of time *F* (*P*)	Main effect of group *F* (*P*)	Post hoc (mean difference, 95% CI)	*P*
TUG (s)	Pre	16.89 ± 14.48	8.77 ± 2.12	4.778 (.036*)	12.072 (.001*)	5.277 (.028*)	8.116 (1.108–15.124)	.025
	Post	13.48 ± 1.52	7.99 ± 1.91				5.483 (.359–1.607)	.037
6MWT (m)	Pre	374.67 ± 167.70	462.06 ± 78.03	.040 (.843)	3.682 (<.001*)	4.123 (.050)	–	–
	Post	415.94 ± 159.35	50.114 ± 83.61					
Gait speed (m/s)	Pre	1.04 ± .47	1.28 ± .22	.039 (.844)	23.938 (<.001*)	4.117 (.050)	–	–
	Post	1.16 ± .44	1.39 ± .23					
5TSTS (s)	Pre	18.01 ± 13.30	11.59 ± 3.66	.475 (.495)	4.251 (.045*)	5.825 (.021*)	–	–
	Post	14.62 ± 7.23	9.95 ± 2.20					

Values are expressed as mean ± standard deviation.

The adjusted α value for post hoc analysis was set at .0125.

5TSTS = 5-times sit-to-stand test, 6MWT = 6-minute walk test, CON = control group, INT = intervention group, TUG = timed up-and-go test.

* *P* <.05.

A significant group × time interaction effect was observed for the TUG (*F* = 4.778, *P* = .036), indicating that the INT group experienced greater improvements in functional mobility over time compared to the CON group. Post hoc analysis with Bonferroni correction identified significant pairwise differences in TUG scores between the 2 groups (mean difference = 8.116, *P* = .025 at baseline; mean difference = 5.483, *P* = .037 at 8 weeks). However, these comparisons did not meet the adjusted significance threshold of α = .0125.

### 3.4. Muscle strength assessment

Table 4 illustrates the effects of the intervention on isokinetic knee extension and flexion peak torque. Significant improvements in muscle strength were observed in both groups following the intervention. There was a main effect of time, with statistically significant increases in extensor and flexor strength, as well as eccentric contraction strength, on both the impaired and unimpaired sides. However, no significant group × time interactions were found for any of the secondary muscle strength outcomes.

**Table 4:** Secondary outcome variables before and after the interventions.

			INT (n = 18)	CON (n = 18)	Group*time interaction *F* (*P*)	Main effect of time *F* (*P*)	Main effect of group *F* (*P*)
Isok 60 d/s E ST (pt)	I	Pre	54.89 ± 17.57	81.89 ± 22.11	.001 (.977)	1.807 (.188)	15.924 (<.001*)
		Post	58.78 ± 22.80	85.61 ± 24.69			
	UI	Pre	72.17 ± 15.69	89.33 ± 2.63	.360 (.552)	.571 (.455)	6.275 (.017*)
		Post	76.94 ± 21.50	88.22 ± 23.67			
Isok 60 d/s F ST (pt)	I	Pre	43.00 ± 16.05	58.50 ± 16.50	.442 (.510)	1.136 (.294)	14.334 (.001*)
		Post	37.00 ± 12.60	57.83 ± 24.74			
	UI	Pre	48.78 ± 17.32	62.22 ± 14.51	.690 (.412)	1.849 (.183)	5.382 (.026*)
		Post	47.22 ± 2.40	55.78 ± 13.95			
Isokinetic End (%)	I	Pre	38.86 ± 17.59	53.07 ± 22.63	2.437 (.128)	5.136 (.030*)	5.514 (.025*)
		Post	4.99 ± 23.34	64.63 ± 21.44			
	UI	Pre	45.16 ± 19.06	62.78 ± 21.44	.396 (.533)	8.178 (.007*)	4.931 (.033*)
		Post	55.90 ± 22.95	69.64 ± 28.03			
Ecc ST (pt)	I	Pre	85.83 ± 24.65	113.61 ± 24.66	.128 (.723)	2.126 (.154)	2.172 (<.001*)
		Post	78.22 ± 2.93	11.72 ± 24.20			
	UI	Pre	92.78 ± 26.11	119.44 ± 19.39	.063 (.804)	2.815 (.103)	16.843 (<.001*)
		Post	85.50 ± 21.02	112.33 ± 23.54			

Values are expressed as mean ± standard deviation.

CON = control group, Ecc ST = eccentric contraction strength, I = impaired side, INT = intervention group, Isok 60 d/s E ST = isokinetic 60 degree/sec extensor strength, Isok 60 d/s F ST = isokinetic 60 degree/sec flexor strength, Isokinetc End = isokinetic muscle endurance, UI = unimpaired side.

* *P* < .05.

### 3.5. Subgroup analysis of the mobility impairment group

Subgroup analysis was conducted for participants with limited walking ability, defined as those who walked <400 m on the 6MWT, referred to as the mobility impairment group. The analysis revealed significant improvements in the TUG and gait speed over time in the INT group (Table 5).

**Table 5:** Subgroup analysis for mobility impairment group.

Factor		Pre (median)	Post (median)	Post–pre (median)	*P*	
					Time	Group
TUG (s)	INT (n = 8)	25.02	16.31	−3.96	.012*	.019*
	CON (n = 5)	1.42	1.19	−.58	.080	
5TSTS	INT (n = 8)	21.00	17.65	−.52	.484	.354
	CON (n = 5)	14.55	12.10	−2.82	.043*	
6MWT (m)	INT (n = 8)	154.00	273.00	34.50	.017*	.943
	CON (n = 5)	388.00	431.00	36.00	.080	
Gait speed (m/s)	INT (n = 8)	.43	.76	.095	.017*	.943
	CON (n = 5)	1.08	1.20	.100	.080	

Values are expressed as median.

5TSTS = 5-times sit-to-stand test, 6MWT = 6-minute walk test, CON = control group, INT = intervention group, TUG = timed up-and-go test.

* *P* < .05.

In the TUG test, the INT group demonstrated a median decrease of 3.96 seconds, which was statistically significant (*P* = .012), while the CON group showed a smaller decrease of 0.58 seconds, which was not significant (*P* = .080). Between-group comparisons revealed a significant difference in post–pre changes (*P* = .019), favoring the INT group. Concerning gait speed, the INT group improved by 0.095 m/s (*P* = .017); the CON group showed a similar improvement of 0.100 m/s, but this was not significant (*P* = .080). The between-group difference in gait speed was not significant (*P* = .943). No significant differences were found in the 5TSTS or 6MWT distances. Both groups showed similar improvements in the 6MWT (INT: 34.5 m, CON: 36.0 m), but neither the time nor group effect reached statistical significance.

## 4. Discussion

Stroke patients often experience significant impairments in daily activities due to spasticity, contracture, and hemiparesis, which are characterized by muscle weakness and reduced motor activity.^[5,11,37]^ Restoring gait function and improving physical performance are primary goals for stroke survivors. Effective rehabilitation programs focus on enhancing functional mobility, endurance, and strength to ultimately improve overall quality of life.

This study assessed the effectiveness of eccentric contraction-based resistance exercise compared to conventional resistance training in chronic stroke patients. The key finding was that both groups demonstrated significant improvements in physical performance measures, including mobility, walking endurance, and gait speed, after 8 weeks of intervention. However, a group × time interaction effect was observed only for the TUG test, indicating that the INT group experienced greater functional improvements over time compared to the CON group. This suggests that while both types of resistance training are beneficial, eccentric-based exercises may offer an additional advantage in enhancing functional mobility in specific aspects. The significant improvement in the TUG test underscores the potential of eccentric training to positively influence functional capacity, particularly in mobility-related tasks. The TUG test, a widely used measure of functional mobility in stroke patients, reflects improvements in balance, agility, and the ability to perform daily activities safely.^[38]^ Gault et al^[39]^ reported that eccentric endurance training through downhill treadmill walking significantly improved TUG scores in older adults, while Fernandez-Gonzalo et al^[22]^ observed similar benefits using a flywheel leg press in chronic stroke patients. These findings support our results, indicating that eccentric exercises may further enhance mobility and balance in stroke survivors.

Our subgroup analysis of participants with mobility impairments highlighted the potential benefits of eccentric training for those with limited walking capacity. The INT group showed significant improvements in TUG and gait speed, while the CON group did not, with between-group comparisons favoring the INT group. Given that walking ≥400 m is crucial for community ambulation,^[40]^ these findings suggest that eccentric training may be particularly effective for stroke patients with severe mobility limitations. Previous studies have shown that eccentric exercises promote muscle regeneration and neuromuscular adaptations, enhancing mobility in stroke survivors while placing a lower demand on the cardiopulmonary system.^[8,19,22,23]^ Our results align with these findings, demonstrating that structured eccentric training can significantly improve functional outcomes in chronic stroke patients, particularly those with mobility impairments.

Consistent with our findings, several studies have reported no significant differences between eccentric and traditional resistance training regarding muscle strength and functional recovery in stroke survivors.^[25,41]^ This suggests that while eccentric exercises offer unique benefits, they may not consistently outperform conventional resistance training, particularly in high-functioning chronic stroke patients. Our results indicate that both eccentric and traditional resistance training significantly improved most physical performance outcomes, but neither approach demonstrated clear superiority. These findings highlight the importance of a structured and tailored exercise regimen, irrespective of the training modality, as a cornerstone of effective stroke rehabilitation.

Both groups demonstrated substantial improvements in walking endurance, as measured by the 6MWT – a widely recognized assessment of walking capacity and endurance that predicts community ambulation in stroke patients.^[33]^ The observed increases in 6MWT distances indicate that both rehabilitation can effectively enhance cardiovascular fitness and endurance. These improvements may enable stroke survivors to walk longer distances with greater ease, thereby promoting better community mobility and improved quality of life. Similarly, the significant improvements in gait speed are important, as they relate to functional independence and fall risk in stroke survivors.^[34,42]^ Both groups showed statistically significant gains, highlighting that regular, structured exercise – eccentric or conventional – effectively enhances walking ability and mobility.

Despite improvements in physical performance, neither group showed significant gains in isokinetic muscle strength after 8 weeks. This may be due to entrenched neuromuscular adaptations in chronic stroke patients, including altered motor unit recruitment and compensatory movement patterns, which can limit strength gains from short-term interventions.^[43–45]^ Additionally, the high baseline functional status of participants, with most achieving maximum scores on the modified Barthel index, may have constrained further improvements, with diminishing returns affecting high-functioning stroke survivors.^[11,37,46]^ These findings suggest that more intensive or prolonged training protocols may be needed to achieve meaningful strength adaptations. Future research should explore higher training volumes, extended intervention periods, or combined approaches to optimize outcomes for this

This study has several limitations. First, the small sample size may have limited the ability to detect subtle differences between eccentric and conventional resistance training, emphasizing the need for larger studies to confirm these findings. Second, the 8-week intervention period may not have been sufficient to capture long-term muscle strength adaptations, particularly in chronic stroke patients with entrenched neuromuscular changes. Future studies should investigate whether extended eccentric training can yield greater strength improvements. Third, variability in baseline characteristics, such as muscle strength and functional capacity, may have influenced the results. Although adjustments were made, larger sample sizes and stratified analyses could provide clearer insights into which stroke patients benefit most from eccentric training. Another limitation of the study is that the intervention group received supervised exercise sessions conducted by an exercise specialist, whereas the control group did not receive equivalent attention. This difference in attention may have influenced the outcomes independently of the intervention itself.

Overall, while both resistance training approaches yielded positive outcomes, eccentric contraction-based resistance training may provide additional benefits for specific subgroups of stroke patients, particularly those with mobility impairments. These findings underscore the importance of tailoring rehabilitation programs to meet the unique needs of each patient, focusing on their functional capacity and walking ability.

## 5. Conclusion

Both eccentric overload and traditional resistance training significantly improved physical performance in chronic stroke patients, highlighting the importance of structured exercise in rehabilitation. While eccentric training may offer additional benefits for those with mobility impairments, both approaches can effectively enhance functional outcomes. Future research should optimize exercise protocols to maximize long-term muscle strength adaptations in this population.

## Author contributions

**Conceptualization:** Jae-Young Lim.

**Data curation:** Seunglyul Oh, Dae Young Kim.

**Formal analysis:** Younji Kim.

**Funding acquisition:** Jae-Young Lim.

**Investigation:** Seunglyul Oh, Dae Young Kim.

**Methodology:** Younji Kim.

**Project administration:** Jae-Young Lim.

**Resources:** Younji Kim.

**Supervision:** Jae-Young Lim.

**Visualization:** Younji Kim.

**Writing – original draft:** Younji Kim, Seunglyul Oh.

**Writing – review & editing:** Younji Kim, Jae-Young Lim.
